# The presence and strength of post-pollination barriers in an orchid lineage that lacks pollinator specialization

**DOI:** 10.1093/aob/mcaf271

**Published:** 2025-10-29

**Authors:** Ilaria Masullo, Donata Cafasso, Maria Rosaria, Maria Rosaria Barone Lumaga, Salvatore Cozzolino

**Affiliations:** Department of Biology, University Federico II of Naples, 80126 Naples, Italy; Department of Biology, University Federico II of Naples, 80126 Naples, Italy; Department of Biology, University Federico II of Naples, 80126 Naples, Italy; Department of Biology, University Federico II of Naples, 80126 Naples, Italy; Department of Biology, University Federico II of Naples, 80126 Naples, Italy

**Keywords:** *Anacamptis*, asymmetric barrier, conspecific pollen precedence, deceptive orchid, orchid seed germination, ovule development, pollen tube growth, pollen–stigma interaction, post-pollination barrier, reproductive isolation

## Abstract

**Background and Aims:**

Post-pollination pre-zygotic barriers involve interactions between the pollen, pistil and ovules that impede fertilization by heterospecific pollen. These barriers act sequentially when pollen from different donors simultaneously pollinates the same flower, by inhibiting the development of foreign pollen or by conferring a fecundation advantage to the specific pollen tube. Here, we investigated the presence and strength of post-pollination barriers in an orchid lineage that lacks pollinator specialization. We then evaluated the relationship between early and late post-pollination pre-zygotic barriers and their chronology of insurgence.

**Methods:**

We conducted hand pollinations by adding conspecific and/or heterospecific pollen to the stigma of the receiving species. From all crosses, we investigated the formation of fruits, the development of the pollen tubes (using scanning and epifluorescence microscopy), the percentage of embryonated seeds, and the seed paternity by molecular analyses of *in vitro* germinated seeds (protocorms) produced in double pollinations.

**Key Results:**

Post-pollination pre-zygotic barriers related to the interaction between the pollen tube and pistil or between the pollen tube and ovule were found to be variable in strength and with strong asymmetry in species pairs. Conspecific pollen precedence (CPP) was found to be strong in most cross combinations: when both conspecific and heterospecific pollen were added simultaneously, conspecific pollen fertilized most of the ovules.

**Conclusions:**

We observed that CPP is stronger than early gametic incompatibility as pollen–pistil incompatibility, despite the latter occurring earlier than CPP. We did not find that species with stronger early gametic incompatibility have less CPP, as expected from a sequential development of these post-pollination barriers. CPP is likely to have existed in the lineage before species separation and has strengthened progressively over the time of species divergence. In contrast, pollen–pistil incompatibilities might have evolved several times and asymmetrically after species divergence, probably prompted by ecological interactions between species during secondary contact.

## INTRODUCTION

The emergence of new species hinges on the gradual acquisition of traits that hinder the successful reproduction of viable and fertile offspring between different populations (Mayr, 1942). These traits, known as reproductive barriers, come in various forms and can be pre- and post-mating based on when they manifest in the life cycle of an organism (Ramsey *et al.*, 2003; Coyne and Orr, 2004). Pre-mating barriers occur before the arrival of heterospecific pollen on the stigma of a species and are exclusively pre-zygotic, whereas post-mating barriers can arise from either pre- or post-zygotic mechanisms (Snow, 1994). Understanding how different barriers contribute to keeping species reproductively isolated and how quickly they develop is a key question in studying how new species form and how existing species survive during secondary contact (Coyne and Orr, 1989; Matute and Cooper, 2021).

In flowering plants, pre-zygotic barriers generally have a stronger effect on reducing gene flow in comparison to post-zygotic barriers, with pre-pollination barriers (i.e. premating isolation) often being predominant in groups with specialized pollination strategies (Christie *et al.*, 2022). After pollination but before ovule fertilization, post-pollination (pre-zygotic) barriers, acting at different stages, can continue to hinder hybridization by involving interactions between heterospecific pollen, pistil and ovule that render foreign pollen incapable of fertilization. Post-pollination barriers can operate as soon as pollen is deposited on a stigma, inhibiting the early development of heterospecific pollen tubes, or later, when the female transmitting tissue can selectively limit the growth and orientation of pollen tubes. Finally, at the ovule level, differential pollen tube attraction and inhibition of sperm cell release can act as barriers to gamete syngamy (Broz and Bedinger, 2021).

The contribution of post-pollination barriers has been investigated widely in plants (reviewed by Rieseberg and Willis, 2007; Christie *et al.*, 2022), although the molecular mechanisms have been identified only in a few, mostly model, species (Broz and Bedinger, 2021; Wang and Filatov, 2023). In some instances, early pollen–pistil interactions at the stigma stage create absolute barriers to cross-species pollination, but in many others, reproductive isolation is intensified through competition between intra- and interspecific pollen (Fishman *et al.*, 2008; Baek *et al.*, 2016), a phenomenon known as conspecific pollen precedence (CPP) (Howard, 1999). It occurs when the stigma of a plant simultaneously receives pollen from the same species and pollen from different species, but the offspring that form are preferably non-hybrid (Hauser *et al.*, 1997; Wolf *et al.*, 2001). In *Mimulus*, analyses of transmission ratio distortion in progeny derived from interspecific pollen mixtures suggest that at least eight genomic regions contribute to CPP. These species-specific differences in pollen tube performance accumulate gradually and might have been driven by intraspecific co-evolution between pollen and style, emphasizing that the female transmitting tissue can selectively limit the growth and orientation of heterospecific pollen tubes (Fishman *et al.*, 2008).

Given that contemporary pollination by conspecific and heterospecific pollen is common in nature, CPP could be a widespread reproductive barrier in plants (Arnold *et al.*, 1993; Howard, 1999; Aldridge and Campbell, 2006; Nista *et al.*, 2015). However, the evolution of strong pollen–pistil interactions, particularly at the stigma stage, can hinder the existence of already existing but later stages of reproductive isolation, such as CPP. Indeed, reproductive barriers continue to evolve even after gene flow ceases between divergent species. This evolution complicates our ability to draw strong conclusions about the pace and importance of the barriers that developed earlier during species divergence (Sobel *et al.*, 2010). Later-evolved barriers might obscure the effects of earlier-evolved barriers, particularly when these later barriers are presently more important for maintaining species boundaries (Coyne and Orr, 1997; Kulmuni *et al.*, 2020).

In some cases, the temporary or partial breakdown of early reproductive barriers (such as when rare hybrids are produced) helps to estimate the presence of later stages of reproductive isolation (such as hybrid sterility or hybrid breakdown) that would otherwise never be observed. Manual pollinations, for example, allowed the estimation of post-pollination barriers (both pre- and post-zygotic) between plant species that are completely isolated by pre-pollination barriers (Moyle *et al.*, 2004; Scopece *et al.*, 2007; Lowry *et al.*, 2008). Likewise, in some plant groups, the application of compatible pollen helps incompatible pollen to overcome the hybridization barrier on the stigma and produce new hybrids that are highly unlikely to occur in nature. Alternatively, compatible heterospecific pollen transfer induces self-pollen germination in otherwise self-incompatible plants. These approaches, known as the pollen mentor effect (Stettler and Ager, 1984), have traditionally been developed for agronomic applications to promote self-pollination or create interspecific hybrids of agronomic or ornamental interest (Lin *et al*., 2025), but have been applied successfully in studies on wild species (Gaget *et al.*, 1989; Desrochers and Rieseberg, 1998).

A lock-and-key molecular model has been validated as the molecular mechanism underlying the pollen mentor effect (Zhong *et al.*, 2025). In Brassicaceae model species, the interaction among various receptor-like kinases, cell-wall proteins and stigmatic rapid alkalinization factor (RALF) peptides (sRALFs) or heterospecific pollen RALF peptides (pRALFs) on the stigma surface governs the penetration of pollen tubes in the pistil and determines the self-incompatibility or the success of hybridization between distant species (Lan *et al.*, 2023).

In orchids, the specialization of pollination strategy has been widely considered the main reproductive barrier and the primary driver of speciation processes (van der Pijl and Dodson, 1966; Dressler, 1993). Consequently, most studies have focused on describing and estimating pre-mating isolation, paying less attention to the role of post-mating (pre- and post-zygotic) barriers (reviewed by Cozzolino and Widmer, 2005). Over the years, however, it has been shown that orchid pollination includes both pollinator-specialized and pollinator-generalized strategies; the latter attract different species of pollinators (bees, butterflies and flies) in a non-selective manner (Cozzolino *et al.*, 2005; Joffard *et al.*, 2019; Ackerman *et al.*, 2023).

Among pollinator-generalized strategies, food-deceptive orchids allure and dupe primarily inexperienced pollinators by mimicking the appearance of typically nectar-producing plants (Dafni, 1987; Jersáková *et al.*, 2009). Their low pollinator specificity does not prevent the risk of incorrect pollen transfer between different species growing in sympatry and having overlapped flowering phenology (Cozzolino *et al.*, 2005). Nonetheless, despite numerous hybrid combinations being described and often reported, true hybrid zones between food-deceptive orchids are surprisingly rare (Dafni, 1987). Typically, they comprise only a few individuals of hybrid origin from early generations (Pellegrino *et al.*, 2005; Moccia *et al.*, 2007), and these hybrids are often poorly fertile (Scopece *et al.*, 2008; Arida *et al.*, 2021), suggesting the presence of strong post-pollination (both pre- and post-zygotic) isolation mechanisms (Cozzolino *et al.*, 2004; Scopece *et al.*, 2007; HP Zhang *et al.*, 2022).

Unlike other angiosperms, ovule development in orchids is triggered by the arrival of conspecific pollen on the stigma (Zhang and O’Neill, 1993; Mayer *et al.*, 2021). Exogenous application of auxin was found to mimic pollination and to initiate most pollination-regulated developmental events, suggesting that the application of artificially synthesized keys to the stigma can function as mentor pollen, facilitating the breakdown of the stigmatic barrier and enabling plant outcrosses (Johansen, 1990).

Lack of a recognition signal (between pollen and stigma) might allow closely related orchid species to share pollen without developing ovules and fruits (Pellegrino *et al.*, 2010; Pellegrino, 2016). Concordantly, comparative studies found that pollen–stigma incompatibility (estimated as fruit development) was stronger in pairs of species with generalized food deception than in pairs of highly specialized sexually deceptive species, i.e. stronger in those species pairs with a higher likelihood of sharing pollinators (Scopece *et al.*, 2007). In the deceptive *Orchis italica* and *O. anthropophora*, even if heterospecific pollen was applied first to the receiving stigma (≤72 h earlier), the pollinated flowers remained receptive to conspecific pollen arrival for a long time, demonstrating, at least in this specific case, that there was an advantage of conspecific pollen for promoting fruit formation, whether it arrived before or after heterospecific pollen (Luca *et al.*, 2015). Despite this evidence, it remains unclear whether heterospecific pollen can compete with conspecific pollen for ovule fertilization and whether this post-pollination barrier was already present in the phylogenetic lineage or has evolved secondarily because of ecological interactions.

Here, we focused on post-mating reproductive isolation among four species of the genus *Anacamptis* (*Anacamptis fragrans*, *A. laxiflora*, *A. morio* and *A. papilionacea*), which are characterized by largely overlapping distribution and pollinators. These four species are considered at the end of the pathway to complete reproductive isolation (RI) and separated ∼9.2 Mya (95 % confidence interval: 6–12 Mya) (Inda *et al.*, 2012). Nonethless, hybrids have been reported seldom, but hybrid swarms are rare, and hybrids are often sterile (Moccia *et al.*, 2007; Scopece *et al.*, 2008). This suggests a strong component of post-pollination isolation (pre- and/or post-zygotic) in maintaining species boundaries in this orchid lineage with low pre-pollination isolation.

In the present study, we carried out hand pollinations in which only conspecific pollen or only heterospecific pollen was added to the stigma of a receiving species and pollinations in which both conspecific and heterospecific pollens (double pollinations) were added simultaneously to the stigma of the same receiving species. Conspecific and heterospecific pollination helped us to identify the presence and strength of early post-pollination pre-zygotic isolation (formation of fruits and ovules). When only heterospecific pollen is placed on the stigma, there is no interspecific pollen competition, and the only variable is the germination of the foreign pollen on the stigma. Instead, adding conspecific pollen (i.e. pollen mentor) to interspecific pollinations (i.e. double pollinations) removes the potential gametic or stigmatic incompatibility that inhibits heterospecific pollen germination and enables the evaluation of pre-zygotic barriers related to pollen tube competition (CPP). From all crosses, we investigated the development of the pollen tubes using scanning and epifluorescence microscopy, and seed paternity by molecular analyses of seeds germinated *in vitro* (protocorms).

Initially, we aimed to distinguish the relative contribution of different stages of post-pollination pre-zygotic barriers (i.e. inhibition of pollen tube development, failure of fruit development, lack of egg fertilization, and conspecific pollen precedence). Then we tested the hypothesis that species pairs with stronger (earlier) pollen–pistil incompatibility have less (later) conspecific pollen precedence and whether these early and late postpollination barriers have concordant or opposite asymmetry within species pairs.

## MATERIALS AND METHODS

### Study system

*Anacamptis fragrans* (Pollini) R.M. Bateman, Pridgeon & M.W. Chase, *A. laxiflora* (Lam.) R.M. Bateman, Pridgeon & M.W. Chase, *A. morio* (L.) R.M. Bateman, Pridgeon & M.W. Chase, *A. papilionacea* (L.) R.M. Bateman, Pridgeon & M.W. Chase and *A. pyramidalis* (L.) Rich. are the most representative members of the five main phyletic lineages of *Anacamptis*. The genus also includes several derivative species with more localized distributions, all of which can be traced back to these five widespread species (Cozzolino *et al.*, 2001; Bateman *et al.*, 2003). Within *Anacamptis*, *A. pyramidalis* represents the only notable exception, also in terms of floral diversification, because it switched to butterfly pollination and acquired strong pollinator isolation from other species, even if occasional pollination by flies and bees was reported. The four remaining investigated species consistently share pollinators (mainly solitary bees, honeybees and bumblebees), have overlapping phenology and co-occur widely. Except for *A. fragrans*, which secondarily acquired the ability to produce nectar (Cozzolino *et al.*, 2001), all *Anacamptis* species use a generalized food deceptive strategy (Dafni, 1987) with different degrees of bee pollinator specialization (mainly bees in *A. papilionacea* and bumblebees in *A. laxiflora*) (van der Cingel, 1995). All plants used for experimental crosses were collected over the last 10–15 years from various natural populations in central and southern Italy and cultivated in pots at the University Campus of Monte Sant’Angelo (Naples, Italy).

### Intraspecific, interspecific and double pollinations

Manual pollination experiments were conducted during the spring in 2020–2022. At flowering time, each plant was covered with a thin nylon netting to prevent access to the flowers by pollinating insects. Between the four species under study, manual pollinations were performed in both directions as follows: (1) intraspecific pollinations; (2) interspecific pollinations; and (3) double pollinations (mixtures of conspecific and heterospecific pollen). All pollinations were conducted by carefully removing the pollinia from freshly opened flowers using forceps and placing them onto the stigmas of other plants of either the same species (intraspecific pollinations) or a different species (interspecific pollinations). for each species pair, we conducted nine intraspecific, nine interspecific and nine double pollinations. In total, we performed 108 interspecific pollinations and 108 double pollinations (4 × 3 species combinations, each ×9 replicates), plus 36 intraspecific pollinations (9 replicates for each of the 4 species). Additionally, we carried out 60 interspecific pollinations (4 × 3 species combinations, each ×5 replicates) and 20 intraspecific pollinations (5 replicates for each of the 4 species) for microscopic observation of pollen tube development. Performing all interspecific pollination treatments on a single plant was unfeasible owing to the risk of resource limitation, the limited number of simultaneously available flowers and the asynchronous flowering pattern. As an additional constraint, when possible, a maximum of three open flowers per plant were used as pollen recipients to minimize the risk of resource limitation, and all plants subjected to pollination treatments (interspecific and double pollinations) received one intraspecific pollination, which was used as a positive control. Then the various pollination combinations planned for each recipient species were not performed on a single plant but were instead randomly distributed across multiple individuals by crossing, on average, 30–40 plants per species, which served both as pollen donor and as pollen recipient, based on flower availability at the time of the experiment. A single pollinium was used in each cross, randomly selected from the available open flowers that also served as the pollen recipient in the reciprocal cross. We always ensured that a comparable number of pollen massulae were added for each pollination. Double pollinations were conducted by applying equal mixtures of conspecific and heterospecific pollen onto the receiving stigma. In any case, we never considered in our analyses the number of seeds per fruit (i.e. seed set), which is dependent on the number of pollen massulae added. Each pollinated flower was marked with a coloured ribbon, and the use of distinct colour codes allowed for clear identification of the different pollination treatments, ensuring accurate tracking during fruit collection. The complete crosses dataset has been submitted to the Dryad public data repository (10.5061/dryad.n2z34tn9j).

The pollination success was determined by comparing the number of fruits produced with the number of pollinated flowers. We collected the ripe fruits resulting from the different types of pollinations (intraspecific, interspecific and double pollination) and stored the seeds at +4 °C. We observed the seeds from each fruit under a compound microscope at 5× magnification and evaluated the presence or absence of embryos in each seed. We examined three replicates of 100 seeds per fruit. For each paired comparison, we performed a Mann–Whitney *U*-test in R v.4.3.3. Then, a representative sample of seeds from fruits produced with double pollinations was germinated *in vitro* and underwent molecular analyses to determine the potential presence of hybrid plants.

### Examination of pollen tube growth and flower stylar length

Additional pollinations were conducted for each species pair combination for microscopic analyses. For this aim, 6 days after manual pollination, the pollinated flowers were excised and softened in 4 m NaOH for 10 min. Subsequently, the stigmas were treated with a 0.05 % solution of Aniline Blue in phosphate buffer (pH 8.3) for 24 h. The samples were then observed under an epifluorescence microscope (Leica M205 FCA, Leica Microsystems, Wetzlar, Germany) with blue light (410 nm) to evaluate pollen germination and observe the development of pollen tubes along the ovary of the pollinated flower. For scanning electron microscope observations, the samples were initially dehydrated using a graded series of ethanol. They were then dried using the critical point method with liquid CO_2_. After drying, the samples were coated with gold/palladium to ∼30 nm and examined using a scanning electron microscope (Nova NanoSEM 450, Thermo Fisher Scientific, Waltham, MA, USA).

In the samples observed under the epifluorescence microscope, the distance from the stigmatic surface to the upper ovules in the ovary (i.e. stylar length) was measured with ImageJ. Then we tested significant differences in stylar length among species with Dunn’s test with Bonferroni correction as implemented in R v.4.3.3.

### *In vitro* asymbiotic germination of seeds

We randomly collected five of the nine mature fruits resulting from each double pollination combination. The seeds produced by these five fruits were then treated with a 10 % NaOCl solution for 15 min to sterilize their surface, following the methods described by Hicks (2000) and Sgarbi *et al.* (2007). This process continued until the seeds appeared bleached. Afterwards, the seeds underwent three rinses with sterile distilled water, with centrifugation at 3500*g* for 5 min between each rinse. BM-1 Terrestrial Orchid Medium (PhytoTechnology Laboratories, Shawnee Mission, KS, USA) was used as a growth medium. The medium was supplemented with 1 % agar and 0.2 % activated charcoal. Given that each orchid fruit can contain ≤1000 dust-like seeds, only ∼100–300 seeds from each fruit were placed in sterile conditions in Petri dishes. Three replicate dishes were set up for each batch of seeds (for a total of 15 dishes for each double-cross combination) to reduce the risk of contamination. The germination dishes were then placed in an incubator at 24 °C in complete darkness (Lauzer *et al.*, 2007). The cultures were examined once a week using a stereoscopic microscope. Upon germination, when the seeds reached the protocorm stage, the plates were exposed to light, with a photoperiod of 16 h light–8 h dark (Magrini *et al.*, 2011). Once the first leaflet had grown, it was sampled and subjected to molecular analysis.

### Molecular analysis of seed progeny

Genomic DNA was extracted from protocorm leaflets according to Doyle and Doyle (1987). The nuclear ribosomal internal transcribed spacer 2 (ITS2) was amplified by PCR as done by Aceto *et al.* (1999), then digested with the selected restriction enzymes following the manufacturer’s instructions (Thermo Fisher Scientific). The sequence of ITS2 for the four *Anacamptis* species, already available in GenBank, was first digested *in silico*. Exclusive restriction enzyme combinations were chosen to cut the ITS2 of the species used as heterospecific pollen and/or as ovule donors (Supplementary Data Fig. S1). Subsequently, the digested fragments were separated by electrophoresis on a 2.5 % agarose gel, alongside a 100 bp ladder used as the molecular weight marker and photographed on an ultraviolet transilluminator.

### Estimates of reproductive isolation

To assess how effectively each reproductive barrier prevents mating between different species and their overall contribution to total reproductive isolation, we adapted a set of metrics developed by Sobel and Chen (2014). These metrics can range from minus one (all matings are interspecific) to one (no interspecific mating), with zero indicating random mating. Therefore, the strength of each reproductive barrier was calculated using derivations of the following equation: RI=1−2x, where the value of *x* represents how likely hybrid gene flow is between species, H denotes the interspecific and C the intraspecific pollination [x=H/(H+C)].

After having averaged the rate of fruits from the nine replicates, for the estimation of pollen–pistil isolation (PPI) we used:


$$RI_{\mathrm{PPI}}=1-2\times \left[\frac{\mathrm{Rate}\;\mathrm{of}\;\mathrm{fruits}\;\mathrm{in}\;\mathrm{interspecific}\;\mathrm{pollination}}{\left(\mathrm{Rate}\;\mathrm{of}\;\mathrm{fruits}\;\mathrm{in}\;\mathrm{interspecific}\;\mathrm{pollination}+\mathrm{Rate}\;\mathrm{of}\;\mathrm{fruits}\;\mathrm{in}\;\mathrm{intraspecific}\;\mathrm{pollination}\right)}\right]$$

For competitive pollen precedence (CPP), we used a modified version of Sobel and Chen’s metrics (Sobel and Chen, 2014) to account for the presence of post-zygotic barriers that reduced the expected number of hybrid embryos in interspecific crosses. In detail, instead of considering the probability of being hybrid as 50 % (as expected when both conspecific and heterospecific pollen fertilize the same number of ovules and generate the same number of embryos), we accounted for the difference in embryonic seed content between conspecific and heterospecific pollinations attributable to the presence of post-zygotic barriers, as:


$$RI_{\mathrm{CPP}}=1-2\times \left[\frac{\left(\frac{\mathrm{Number}\;\;\mathrm{of}\;\mathrm{hybrids}\;\mathrm{observed}}{\mathrm{Number}\;\mathrm{of}\;\mathrm{hybrids}\;\mathrm{expected}}\right)}{\left(\frac{\mathrm{Number}\;\mathrm{of}\;\mathrm{non}\text{-}\mathrm{hybrids}\;\mathrm{observed}}{\mathrm{Number}\;\mathrm{of}\;\mathrm{non}\text{-}\mathrm{hybrids}\;\mathrm{expected}}\right)+\left(\frac{\mathrm{Number}\;\mathrm{of}\;\mathrm{hybrids}\;\mathrm{observed}}{\mathrm{Number}\;\mathrm{of}\;\mathrm{hybrids}\;\mathrm{expected}}\right)}\right]$$

The existence of complete pollen–pistil isolation in certain species pairs (such as when *A. laxiflora* is pollinated with heterospecific pollen from either *A. papilionacea* or *A. morio*, which led to the absence of fruit formation in these crosses) prevented us from calculating CPP based on the number of embryonic seeds resulting from heterospecific pollinations. In these specific instances, we determined the CPP by using the number of embryonic seeds from reciprocal heterospecific crosses (Supplementary Data Table S1).

The strength of reproductive isolation might vary depending on which species is used as the pollen parent and which is the ovule parent (Tiffin *et al.*, 2001). To explore these variations, we compared the strength of reproductive barriers in reciprocal crosses. We measured the asymmetry of each barrier as the absolute difference in strengths between the two crossing directions (Lowry *et al.*, 2008; Sánchez-Guillén *et al.*, 2012). We performed Spearman’s correlation analysis (*r*_s_; a non-parametric test) in R v.4.3.3 to assess whether there was a monotonic relationship between RI_PPI_ and RI_CPP_.

## RESULTS

### *Anacamptis papilionacea* as ovule (seed) parent

**Fig. 1.**
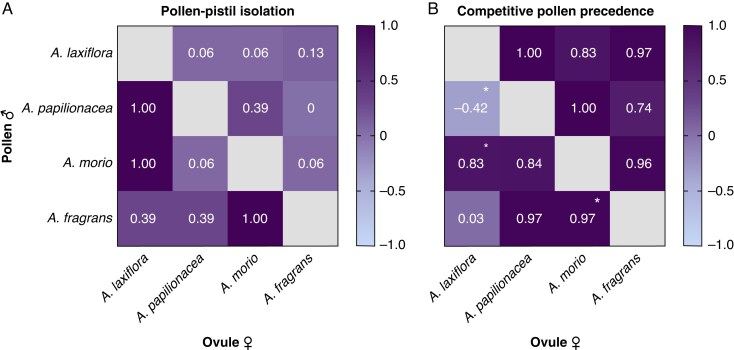
Strength of post-pollination reproductive isolation among species pairs of *Anacamptis*. Two components of pre-zygotic post-pollination isolation are considered: (A) pollen–pistil; and (B) conspecific pollen precedence (CPP). Higher values, shown by darker shaded cells, indicate stronger isolation and a lower likelihood of genetic exchange. Values close to one indicate nearly complete isolation, whereas lower values suggest less effective barriers. An asterisk indicates the crosses whose CPP index has been calculated considering reciprocal crosses.

All intraspecific pollinations resulted in fruit formation. In contrast, in interspecific pollinations, when *A. papilionacea* was pollinated with heterospecific pollen from the other three species, a decrease in fruit formation was observed (Table 1; Fig. 1A). However, in interspecific pollinations, the percentage of seeds containing embryos has always been significantly lower than that in intraspecific pollinations (Table 1). When *A. papilionacea* was pollinated with heterospecific pollen, proper development of pollen tubes along the flower’s ovary was observed. When mixtures of conspecific and heterospecific pollen (of *A. laxiflora*, *A. morio* or *A. fragrans*) were applied to the stigma, fruit formation occurred successfully in all cases. However, when mixtures of conspecific and heterospecific pollen of *A. laxiflora* or *A. morio* were applied to the same stigma, the percentage of embryo-containing seeds produced was significantly higher than that obtained from interspecific pollinations and not significantly different from that of intraspecific pollinations (Table 1). Instead, when mixtures of conspecific and heterospecific pollen of *A. fragrans* were applied to the same stigma, the percentage of embryo-containing seeds produced was not significantly different from that of interspecific pollinations and was significantly lower than that of intraspecific pollinations (*P* = 0.004). By analysing individual protocorms obtained by *in vitro* seed germination, the ITS sequence of the heterospecific pollen donor was detected in a fraction of seeds from some double pollinations (Table 1).

**Table 1.** Reproductive output is presented as fruit set (percentage of fruits per flower pollinated) (A) or as viable seed set (percentage of embryonated seeds per fruit per cross type, ± s.d.) for single pollination (B) or double pollinations (C) across the four listed species; and (D) the number of hybrid protocorms detected relative to the total number of protocorms analysed.

	*A. papilionacea* ♂	*A. laxiflora* ♂	*A. morio* ♂	*A. fragrans* ♂	
***A. papilionacea*** ♀	100.00	88.89	88.89	44.44	A
	84.62 ± 11.69	7.10 ± 7.40 (*P* = 0.001)	51.58 ± 29.97 (*P* = 0.021)	25.75 ± 19.86 (*P* = 0.005)	B
	–	71.66 ± 16.79 (*P* = 0.001)	93.83 ± 5.32 (*P* = 0.001)	48.18 ± 28.58	C
	–	0/151	18/150	1/155	D
***A. laxiflora*** ♀	0.00	100.00	0.00	44.44	A
	N.A.	75.30 ± 7.75	N.A.	25.66 ± 20.34 (*P* = 0.005)	B
	68.98 ± 21.10	–	72.32 ± 12.57	43.11 ± 19.58	C
	31/153	–	19/152	51/156	D
***A. morio*** ♀	44.44	88.89	100.00	0.00	A
	84.40 ± 1.36	45.82 ± 19.90 (*P* = 0.002)	82.84 ± 15.88	N.A.	B
	76.95 ± 16.72	73.44 ± 16.08 (*P* = 0.005)	–	62.97 ± 31.52	C
	8/156	16/156	–	2/152	D
***A. fragrans*** ♀	100.00	77.78	88.89	100.00	A
	37.07 ± 20.68 (*P* = 0.004)	34.95 ± 26.49 (*P* = 0.013)	35.58 ± 18.17 (*P* = 0.005)	69.37 ± 18.53	B
	56.07 ± 22.98	43.18 ± 27.09	57.74 ± 24.00 (*P* = 0.027)	–	C
	22/153	2/150	3/150	–	D

Missing or inapplicable values are indicated as ‘N.A.’. The *P*-values, obtained from Mann–Whitney *U*-tests, indicate in B cases where interspecific pollinations resulted in a significantly lower percentage of embryonated seeds compared with intraspecific pollinations, and in C cases where double pollinations resulted in a significantly higher percentage of embryonated seeds compared with interspecific pollinations.

### *Anacamptis laxiflora* as ovule (seed) parent

All intraspecific pollinations resulted in fruit formation. However, in interspecific pollinations, a decrease in fruit formation was observed when *A. laxiflora* was pollinated with heterospecific pollen from *A. fragrans*. When *A. laxiflora* was pollinated with heterospecific pollen of *A. papilionacea* or *A. morio*, fruit formation was never observed (Table 1; Fig. 1A).

**Fig. 2.**
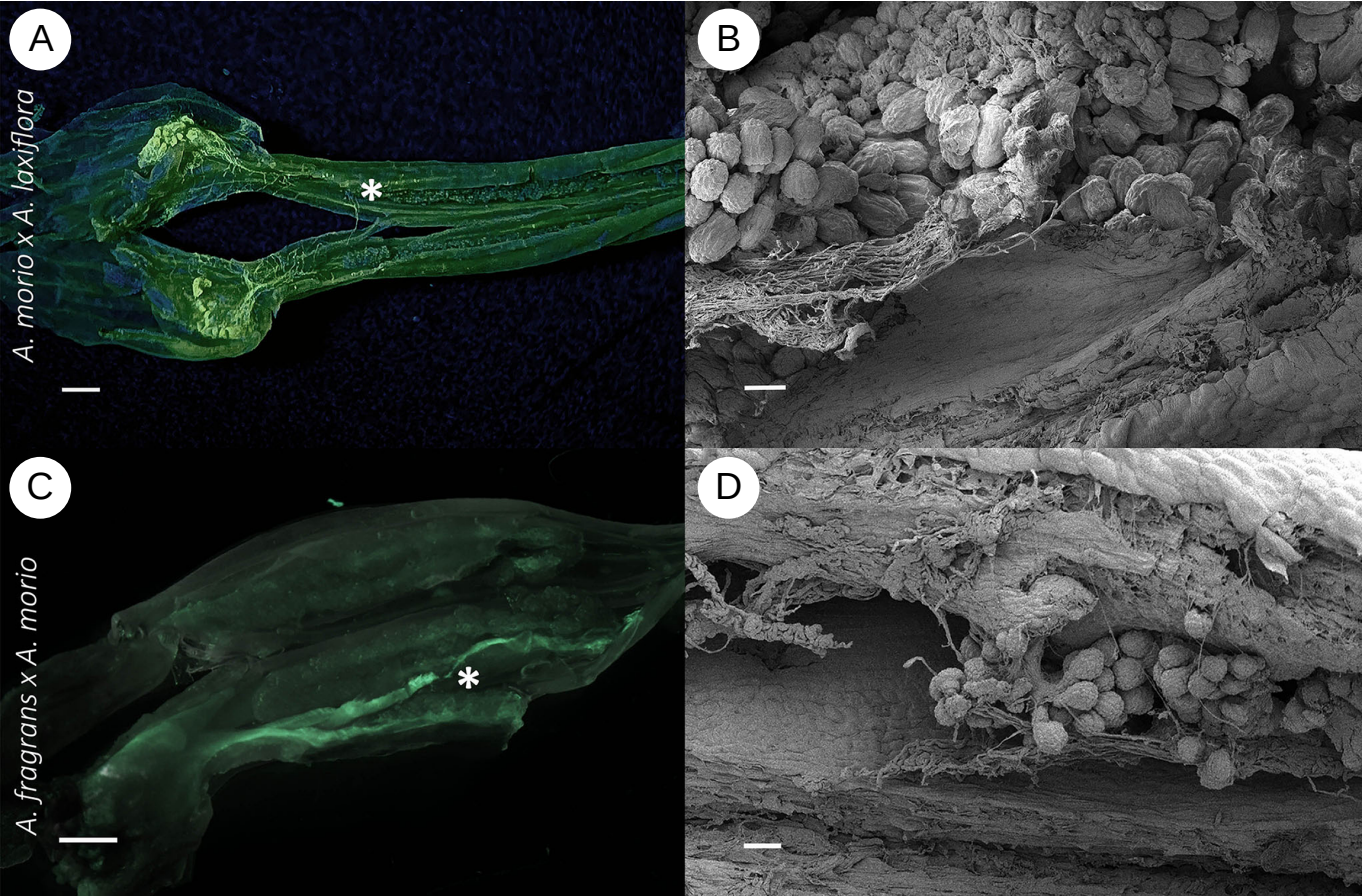
(A) Ultraviolet light picture of the ovary of *Anacamptis laxiflora* pollinated by *Anacamptis morio*, showing the development of heterospecific pollen tubes. (B) Detail (scanning electron microscope picture) corresponds to the area (*) where pollen tubes stop developing without interacting with the undeveloped ovules. (C) Ultraviolet light picture of the ovary of *A. morio* pollinated by *A. fragrans*, showing the heterospecific pollen tubes developed throughout the ovary. (D) Detail (scanning electron microscope picture) corresponds to the area (*) where the pollen tubes interact with the ovules. Several ovules have developed inner and outer integuments incompletely, as in unpollinated flowers. In all pictures, the stigma is on the left side. Scale bars A and C: 1 mm; B and D: 100 μm.

In interspecific pollinations with *A. fragrans* as the pollen donor, the percentage of embryo-containing seeds was significantly lower than that of intraspecific pollinations (Table 1). The microscopic observation of flowers pollinated with heterospecific pollen confirmed the findings obtained from the analysis of fruits resulting from interspecific pollination. When *A. laxiflora* was pollinated with heterospecific pollen from either *A. papilionacea* or *A. morio*, there was an initial development of the pollen tubes. However, only a few massulae developed pollen tubes, and those did not elongate towards the basal part of the ovary as expected. Instead, they grew in various directions and became blocked or tangled, typically remaining confined at the uppermost part of the ovary, without triggering fruit formation (Fig. 2A, B).

Instead, when mixtures of conspecific and heterospecific pollen (of *A. papilionacea*, *A. morio* or *A. fragrans*) were applied to the stigma, fruit formation occurred successfully in all cases.

When mixtures of conspecific and heterospecific pollen of *A. fragrans* were applied to the same stigma, the percentage of embryo-containing seeds was not significantly different from that of interspecific pollinations and was significantly lower than intraspecific pollinations (*P* = 0.004). Instead, when mixtures of conspecific and heterospecific pollen of *A. papilionacea* or *A. morio* were applied to the same stigma, the percentage of embryo-containing seeds was not significantly different from intraspecific pollinations. By analysing individual protocorms obtained by *in vitro* seed germination, the ITS sequence of the heterospecific pollen donor was detected in a fraction of the seeds produced by all double pollinations (Table 1).

### *Anacamptis morio* as ovule (seed) parent

All intraspecific pollinations resulted in fruit formation. In interspecific pollinations, when *A. morio* was pollinated with heterospecific pollen from *A. laxiflora* or *A. papilionacea*, a decrease in fruit formation was observed, and when *A. morio* was pollinated with heterospecific pollen of *A. fragrans*, fruit formation was never observed (Table 1; Fig. 1A).

In interspecific pollinations, with pollen of *A. laxiflora*, the percentage of embryo-containing seeds was always significantly lower than that from intraspecific pollinations (Table 1). In contrast, with the heterospecific pollen of *A. papilionacea*, the percentage of embryo-containing seeds produced was not significantly different from that of intraspecific pollinations.

The microscopic observation conducted on *A. morio* flowers pollinated with heterospecific pollen of *A. fragrans* confirmed the findings obtained from the analysis of fruits resulting from interspecific pollination. When *A. morio* was pollinated with heterospecific pollen from *A. fragrans*, there was a seemingly proper development of pollen tubes. However, even when those pollen tubes elongated well inside the ovary and reached the ovule, they failed to trigger ovule and fruit development (Fig. 2C, D).

When mixtures of conspecific and heterospecific pollen (of *A. laxiflora*, *A. papilionacea* or *A. fragrans*) were applied to the stigma, fruit formation occurred successfully in all cases. However, when mixtures of conspecific and heterospecific pollen of *A. laxiflora* were applied to the same stigma, the percentage of embryo-containing seeds produced was significantly higher than that obtained with interspecific pollinations (Table 1) and was not significantly different from that obtained with intraspecific pollinations. When mixtures of conspecific and heterospecific pollen of *A. papilionacea* were applied to the same stigma, the percentage of embryo-containing seeds produced was not significantly different from that obtained with interspecific pollination and with intraspecific pollination. Finally, when mixtures of conspecific and heterospecific pollen of *A. fragrans* were applied to the same stigma, the percentage of embryo-containing seeds produced was not significantly different from that obtained with intraspecific pollinations. By analysing individual protocorms obtained by *in vitro* seed germination, the ITS sequence of the heterospecific pollen donor was detected in a fraction of the seeds produced by all double pollinations (Table 1).

### *Anacamptis fragrans* as ovule (seed) parent

All intraspecific pollinations resulted in fruit formation. In interspecific pollinations, when *A. fragrans* was pollinated with heterospecific pollen from *A. papilionacea*, fruit formation always occurred. However, with heterospecific pollen from *A. morio* or *A. laxiflora*, a decrease in fruit formation was observed (Table 1; Fig. 1A).

In interspecific pollinations, the percentage of embryo-containing seeds produced was always significantly lower than that of intraspecific pollinations (Table 1). When mixtures of conspecific and heterospecific pollen (of *A. papilionacea*, *A. morio* or *A. laxiflora*) were applied to the stigma, fruit formation occurred successfully in all cases. When mixtures of conspecific and heterospecific pollen of *A. papilionacea* were applied to the same stigma, the percentage of embryo-containing seeds produced was not significantly different from that obtained with interspecific pollinations and intraspecific pollinations. Instead, when mixtures of conspecific and heterospecific pollen of *A. morio* were applied to the same stigma, the percentage of embryo-containing seeds produced was significantly higher than that obtained with interspecific pollinations (Table 1) and was not significantly different from that obtained with intraspecific pollinations. Finally, when mixtures of conspecific and heterospecific pollen of *A. laxiflora* were applied to the same stigma, the percentage of embryo-containing seeds produced was not significantly different from those obtained with interspecific pollinations and was significantly lower than that obtained with intraspecific pollinations (*P* = 0.038). By analysing individual protocorms obtained by *in vitro* seed germination, the ITS sequence of the heterospecific pollen donor was detected in a fraction of the seeds produced by double pollinations (Table 1).

### Asymmetric isolation and flower stylar length

**Fig. 3.**
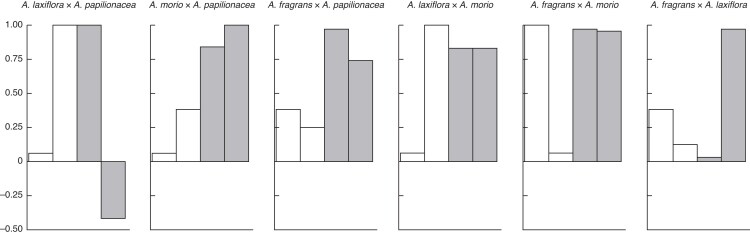
Bar chart illustrating the asymmetry in the strength of pre-zygotic post-pollination isolation in reciprocal crosses. The bars, one for each crossing direction, represent the two categories of post-pollination isolation: pollen–pistil isolation (PPI; white bars) and conspecific pollen precedence (CPP; grey bars).

Pollen–pistil incompatibility emerged as having the most pronounced difference (asymmetry) between species (general mean = 0.73) in comparison to CPP (general mean = 0.38) (Fig. 3). We did not find a significant correlation between the strength of RI_PPI_ and RI_CPP_ (*r*_s_ = −0.07, *P* = 0.84; Supplementary Data Fig. S2). Significant differences were found between *A. fragrans* and *A. papilionacea* (*P* = 0.03) and between *A. morio* and *A. papilionacea* (*P* = 0.02) in stylar length (Supplementary Data Fig. S3).

## DISCUSSION

### The contribution of post-pollination barriers to the reproductive isolation of diverged *Anacamptis* species

Pollinator isolation as a consequence of floral specialization has been considered a primary driver of orchid diversification and the predominant form of species isolation, with the contribution of late-acting barriers largely neglected (van der Pijl and Dodson, 1966; Gaskett, 2011). This general rule has been confirmed several times when estimating isolation in orchid sister pairs with specialized pollinators (Scopece *et al.*, 2007; Xu *et al.*, 2011; Whitehead and Peakall, 2014; Sun *et al.*, 2015) but has been contradicted in studies on orchids with less specialized pollination (Scopece *et al.*, 2008, 2013; Marques *et al*., 2014; Zhang and Gao, 2017; Arida *et al.*, 2021).

Although pollen–pistil incompatibility was expected (and found; see also Scopece *et al.*, 2007) to be strong in *Anacamptis* (i.e. stronger than in orchids with specialized pollinators), here we also investigate the presence and strength of CPP, a neglected aspect of orchid post-pollination pre-zygotic isolation. In particular, we address the interplay between pollen–pistil incompatibility and CPP in *Anacamptis* species, because current estimates of reproductive isolating barriers might not represent it accurately (Widmer *et al.*, 2009). The presence and strength of late-acting barriers (such as CPP) can be concealed by the emergence of early-acting barriers (such as pollen–pistil incompatibility). Differently from incipient species, more diverged species, such as the four *Anacamptis* species in our study, are today separated by barriers important to maintaining species boundaries that were not necessarily present soon after divergence (Coyne and Orr, 1997). Because reproductive isolation might continue to evolve after the cessation of gene flow and because early-acting barriers have a relevant impact on the interruption of gene flow, early-acting barriers, when sufficiently strong, can outshine later-acting ones, regardless of the order in which they evolved (Kulmuni *et al.*, 2020). Thanks to the pollen mentor effect in the double pollinations, we removed the (earlier) post-pollination pre-zygotic barrier (attributable to pollen–stigma incompatibilities) and estimated the presence and strength of CPP isolation even in those species pairs where this late post-pollination barrier was hidden by a strong (complete) early-acting barrier. At the same time, double pollinations allowed the understanding that remaining post-mating reproductive isolation was not complete even between species pairs (today) fully separated by an early post-pollination isolation. This might also account for the existence of natural hybrids even between those species pairs separated by almost complete pollen–stigma incompatibility. The natural occurrence of double pollinations attributable to pollinator sharing between sympatric *Anacamptis* species (as reported by Cozzolino *et al.*, 2005) might remove even the strongest post-mating isolation, allowing the production of a few viable hybrids, as also reported for *Helianthus* (Desrochers and Rieseberg, 1998). Post-pollination barriers are particularly important in plants with generalized pollination (Rieseberg and Willis, 2007; Widmer *et al.*, 2009), and in spite of the disproportionate emphasis on the role of orchid pollination as an isolating barrier, orchids with low pre-pollination isolation do not represent a notable exception to this general trend (Cozzolino and Scopece, 2008).

### The type and strength of pollen–pistil isolation

Pollen–pistil isolation is one of the most important barriers in flowering plants, serving as a mechanism to prevent both self-fertilization and interspecific hybridization (Wheeler *et al.*, 2001; Broz and Bedinger, 2021; Christie *et al.*, 2022). Accordingly, it has been identified as a major component of reproductive isolation between several species pairs of flowering plants (Baek *et al.*, 2016; Liang *et al.*, 2018). However, the uniqueness of orchid flowers, which are primitively self-compatible, and the delayed development of orchid ovules raise questions about whether the same or similar isolating mechanisms might also operate within this plant family.

We have found that the pollen–pistil barrier represents a relevant component of reproductive isolation in *Anacamptis*. Pollen–stigma interactions are important from initial pollen adhesion to fertilization (Twell, 2006) and act in a species-specific fashion (Swanson *et al.*, 2004), suggesting that they could be a major source of pollen–pistil barriers (Broz and Bedinger, 2021). The barrier completely prevents hybridization between species when *A. laxiflora* is pollinated with heterospecific pollen from either *A. papilionacea* or *A. morio*, because fruit formation has never been observed in these crosses. These results were supported by microscopic observation, which revealed that even 6 days after pollination, heterospecific pollen (from *A. morio* and *A. papilionacea*), although germinating, largely remains confined at the stigma level, suggesting the presence of strong stigmatic incompatibility between the pollen of these two species and the stigma of *A. laxiflora*, preventing elongation of pollen tubes deeper into the ovary and the development of fruit (and ovules).

After the arrival of pollen from a different species on the stigma, compounds on the pollen and stigma surface create the adhesion necessary for pollen germination and pollen tube development (Broz and Bedinger, 2021; Wang and Filatov, 2023). This process can be disrupted during pollination between different species (Swanson *et al.*, 2004), suggesting that the pollen–stigma regulatory system might provide a platform for the establishment of reproductive barriers. Pollen–pistil incompatibility also completely prevents hybridization when *A. morio* is pollinated with the heterospecific pollen of *A. fragrans*. Contrary to what was observed in *A. laxiflora*, microscopic observation revealed that when *A. morio* is pollinated with pollen from *A. fragrans*, the heterospecific pollen germinates, and pollen tubes develop all along the ovary but fail to fertilize the ovules (and fruits do not develop). This indicates a strong gametic incompatibility between the heterospecific pollen tubes (from *A. fragrans*) and the ovules of the receiving flower (*A. morio*). Lack of ovule targeting might depend on the species-specific pollen tube–ovule communication, because the attraction of pollen tubes to the embryo sac and ovules often occurs in a species-specific manner (Higashiyama *et al.*, 2006; Takeuchi and Higashiyama, 2012; Lindner *et al.*, 2015; Tao *et al.*, 2018).

Overall, from the results obtained from interspecific crosses, it emerged that in the aforementioned three cases (*A. morio*/*A. papilionacea* × *A. laxiflora*; *A. fragrans* × *A. morio*), a robust (and asymmetric) pollen–pistil barrier between species exists and is related to the interaction between the pollen tube and stigma or between pollen tubes and ovules, whereas successful fruit and seed formation occurs in the majority of other crosses. It is likely that this strongly asymmetrical pollen–pistil barrier evolved only later in *A. laxiflora* and *A. morio* (i.e. after the separation of incipient species), because it is not shared with other species, and it is also clear that two isolating mechanisms (pollen tube elongation and pollen tube–ovule interaction) have evolved independently in these two species.

The addition of conspecific pollen nullifies gametic incompatibilities between heterospecific pollen and stigma or ovules. When *A. laxiflora* was pollinated with both conspecific and heterospecific pollen (from *A. papilionacea* or *A. morio*), fruits formed and contained hybrid seeds. Likewise, in *A. morio*, hybrid-origin seeds were observed when it was pollinated with both conspecific and heterospecific pollen from *A. fragrans*. Therefore, the addition of conspecific pollen, in addition to heterospecific pollen, highlights that in these interspecific pollinations the pre-zygotic barrier is indeed determined by gametic incompatibility between the heterospecific pollen tube and the stigma/ovule of *A. laxiflora* and *A. morio*, respectively (and overcome by the addition of conspecific pollen). The addition of both compatible and incompatible pollen on the same stigma revealed a typical pollen mentor effect (Stettler and Ager, 1984), wherein normally incompatible pollen grains were not able to penetrate the style, leading to speculation that factors in compatible pollen suppress some incompatible barriers, allowing heterospecific pollen tube growth (Knox *et al.*, 1987; de Nettancourt, 2012).

Our results suggest that a self-recognition system (instead of self-incompatibility) exists in *Anacamptis*. Self-incompatibility has evolved secondarily only in some species-rich tropical clades (e.g. *Dendrobium*) (Pinheiro *et al.*, 2015; X Zhang *et al.*, 2021). Instead, the majority of orchids, and all *Anacamptis* species, are self-compatible, and pollinaria bending is the sole mechanical barrier to self-fertilization in nature (Johnson and Edwards, 2000). The mechanism underlying the pollen mentor effect is driven either by the activation of a system required for pollen tube germination or by the deactivation of inhibitors of pollen tube development and growth along the ovary (Broz and Bedinger, 2021). Wet stigmas, as in orchids, generally tend to hydrate pollen indiscriminately, promoting pollen germination (Hiscock and Allen, 2008), as we found in all our crossing combinations. More likely, the addition of conspecific pollen activates pistil systems that promote pollen tube development and orientation (as in *A. laxiflora*) and the interaction between heterospecific pollen tubes and ovules (as in *A. morio*).

### The contribution of conspecific pollen precedence to species isolation

The relevant contribution of CPP to reproductive isolation has been reported among several flowering plants, such as sunflowers (Rieseberg *et al.*, 1995), *Iris* (Carney *et al.*, 1996; Emms *et al.*, 1996), *Mimulus* (Diaz and Macnair, 1999; Ramsey *et al.*, 2003), *Senecio* (Chapman *et al.*, 2005) and *Betula* (Williams *et al.*, 1999), and between diploid and tetraploid populations of *Chamerion* (Husband *et al.*, 2002), but it has been investigated only marginally in orchids (Pellegrino *et al.*, 2010; Scopece *et al.*, 2013). In our study, double pollinations, where both conspecific and heterospecific pollen were applied simultaneously to the stigma of a species, allowed for discerning between possible post-pollination pre-zygotic barriers based on pollen–pistil incompatibility (inhibition of pollen tube development, failure of fruit development or failure of ovule fertilization) and those based on pollen competition or CPP. The latter was found to be strong in almost all crosses and directions: when both conspecific and heterospecific pollen were added simultaneously, it was the conspecific pollen that fertilized most ovules. Despite some small but significant differences in species style length (Supplementary Data Fig. S3), we never observed unilateral pollen precedence in interspecific crosses of species with varying style lengths. This means that no one species consistently demonstrated competitive superiority, regardless of the recipient species (Emms *et al.*, 1996; Aldridge and Campbell, 2006). We also observed that double pollinations with more embryonic seeds were often associated with high CPP values. Indeed, if percentages of embryonic seeds from double pollinations are similar to those from intraspecific pollination and significantly higher than those from interspecific pollination, then it is expected that such a difference is attributable to preferential ovule fecundation by conspecific pollen (i.e. strong CPP) (Table 1; Supplementary Data Table S1).

It is important to note that when estimating the strength of CPP, the differences between non-hybrid and hybrid progeny arise from both CPP and post-zygotic isolation. Indeed, the addition of conspecific pollen does not remove the presence of interspecific post-zygotic barriers that also lead to a reduction in the number of hybrids. The presence of post-zygotic barriers in almost all cross combinations (as confirmed in interspecific crosses) can lead to an overestimation of the strength of CPP. In our calculation of CPP, we considered the difference in embryonic seeds produced through conspecific versus heterospecific pollination. However, we recognize that the number of hybrid progeny observed can be influenced by various stages of early post-zygotic isolation, including seed germination and protocorm development. These potential barriers can lead to an additional overestimation of CPP that current experiments cannot evaluate. For example, in the case of double pollinations involving *A. papilionacea*, when it was pollinated with pollen from both its own species and that of *A. laxiflora*, none of the analysed protocorms was hybrid (and the resulting CPP was 1.00). However, the low percentage of embryonic seeds (only 7.1 ± 7.4 %) produced from the heterospecific pollination of *A. papilionacea* with *A. laxiflora* pollen indicates a very strong post-zygotic barrier. Such a barrier can have a stronger impact on the number of hybrid offspring than CPP.

The consistent observation of reciprocal CPP, whereby the pollen of each species exhibits a competitive advantage on its styles (e.g. Rieseberg *et al.*, 1995), suggests that the speed of pollen tube growth might be influenced greatly by the stylar context. The co-evolution of genes involved in the pollen (such as those related to signalling) and style (including guidance mechanisms) would result in a dependence of pollen performance on the style in interspecific crosses. This is especially likely if a compatible interaction between pollen and style proteins is essential for recognizing and fully nourishing conspecific pollen tubes. In this case, CPP could reflect a history of co-evolution between the pollen and the stylar (or ovular) proteins that are essential for guiding pollen tubes, supporting their growth and facilitating successful fertilization (Swanson *et al.*, 2004).

### The interplay between the different stages of post-pollination isolation and their contribution to reproductive isolation

It is difficult to determine the order of evolution of reproductive barriers (Rieseberg and Willis, 2007), particularly in already diverged species with strong reproductive isolation (Kulmuni *et al.*, 2020). Contrary to expectation, we did not find that species with stronger pollen–pistil incompatibility have less pollen competition or conspecific pollen precedence, as expected from a sequential development of these post-pollination barriers. Overall, with all caveats acknowledged, we found that CPP is generally stronger and more common than pollen–pistil incompatibility, even if this barrier occurs earlier.

It is likely that a barrier such as CPP, now partly hidden by pollen–stigma incompatibilities, had evolved before the emergence of this earlier-occurring barrier. CPP could have been present already in the phyletic lineage (i.e. in the common ancestor) and progressively strengthened with increasing genetic divergence, because we consistently found an advantage of conspecific pollen in almost all crossing combinations, whereas pollen–stigma incompatibilities might have (independently) evolved after species separation. After allopatric speciation, pre-mating isolation might be influenced by natural selection in secondary contact zones (Moyle *et al.*, 2004; Ortiz-Barrientos *et al.*, 2009). Owing to the observed sterility of hybrids in *Anacamptis* (Moccia *et al.*, 2007; Scopece *et al.*, 2008), a strong selection for reducing pollen waste in unfruitful interspecific mating is expected when already diverged species meet in secondary contact. It is likely, therefore, that the lack of pollinator specialization, leading to high levels of pollinator sharing in contact zones among related species (Cozzolino *et al.*, 2005), potentially imposed strong pressure on the evolution of post-pollination barriers for reducing the waste of gametes by production of maladapted hybrids (Cozzolino and Scopece, 2008). These ecological interactions (between non-sister species, as in our case study) might have fuelled, in some species combinations, the insurgence of the pollen–stigma isolating barriers in a manner that is not correlated to the strength of other (later) barriers (Yost and Kay, 2009). According to other plant studies, we found that pollen–stigma incompatibility has strong asymmetric patterns between species pairs (Christie *et al.*, 2022), and this asymmetry does not correspond to concordant or opposite patterns of asymmetry in CPP for the same species pairs (Fig. 3). We argue that the appearance of a pollen–stigma barrier is more closely associated with the ecological history of individual species, whereas CPP is likely to reflect overall genome-wide divergence (Widmer *et al.*, 2009; Garlovsky *et al.*, 2024).

It emerged that in almost all cases, the combined action of both barriers is necessary to achieve the observed strong post-pollination isolation in *Anacamptis*. Total reproductive isolation between the species is robust, but excluding a few crossing combinations, post-pollination isolation is very strong but not complete. This might explain the presence of some hybrids, which is less than expected given the abundance of shared pollinators and that, owing to the strong asymmetry of pollen–stigma incompatibilities, they often have one preferred parental species as ovule donor (Ren *et al.*, 2014).

Even if pre-zygotic post-pollination isolation is effective in maintaining complete reproductive isolation in certain crossing combinations, it might not have been a significant factor during the initial stages of *Anacamptis* species separation, and it might be strengthened because of the low pollinator specificity (i.e. low pre-pollination isolation) typical of these orchids. Accordingly, in the phylogenetically related and highly pollinator-specialized genus *Ophrys*, a significantly lower amount of post-pollination pre-zygotic isolation was found (Scopece *et al.*, 2007). Comparative studies on related lineages with varying pollination strategies could provide valuable insights into the relationship between the degree of pollinator specialization and the evolution and strength of post-pollination barriers in orchids.

## Supplementary Material

mcaf271_Supplementary_Data

## SUPPLEMENTARY DATA

Supplementary data are available at *Annals of Botany* online and consist of the following. Figure S1: table showing all the cross combinations among *Anacamptis fragrans*, *A. morio*, *A. papilionacea* and *A. laxiflora*. The restriction enzyme used to detect hybrid protocorms by cutting the ITS2 sequence is shown for each cross. The coloured segments illustrate the ITS2 region of each species, and the number of enzyme cuts is shown visually. Figure S2: scatterplot showing the relationship between the two stages of post-pollination isolation. The *x*-axis represents pollen–pistil reproductive isolation (RI_PPI_), while the *y*-axis indicates conspecific pollen precedence reproductive isolation (RI_CPP_). Each point represents a different cross. Figure S3: boxplot showing the style length (in millimetres) for the four species: *A. laxiflora*, *A. papilionacea*, *A. morio* and *A. fragrans*. Each box represents the length distribution with the median (central line), interquartile range (upper and lower edges of the box) and extreme values (whiskers). Black horizontal lines and asterisks indicate significant differences between species pairs, based on Dunn’s test with Bonferroni correction: **P* ≤ 0.05. Table S1: the spreadsheet estimates the conspecific pollen precedence (CPP) index related to post-zygotic isolation by comparing the observed proportions of hybrid protocorms with the expected proportions as calculated from the percentage of embryonic seeds in interspecific and intraspecific pollinations. Each row represents a specific combination of pollen donor and ovule recipient.
