# Supplementary material for: The presence and strength of post-pollination barriers in an orchid lineage that lacks pollinator specialization
Source: Ann Bot. 2025 Oct 29;138(2):387–99. doi: 10.1093/aob/mcaf271 (PMC13454692; doi:10.1093/aob/mcaf271)
Supplement: Supplementary file 1 [file mcaf271_supplementary_data.zip › Supplementary figures 1-3.pdf]

**Supplementary Information**

# The interplay of early and late post-pollination pre-zygotic barriers in an orchid lineage with low pre-pollination isolation

Ilaria Masullo, Donata Cafasso, Maria Rosaria Barone Lumaga, and Salvatore Cozzolino*

*Department of Biology, University Federico II of Naples, Naples, Italy*

**For correspondence. E-mail: cozzolin@unina.it*

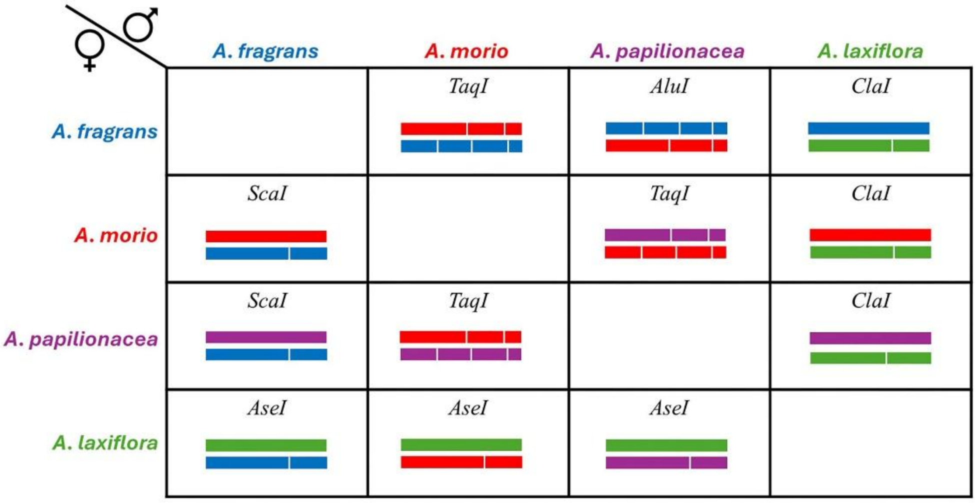


Fig. S1. Table showing all the cross combinations among *A. fragrans*, *A. morio*, *A. papilionacea*, and *A. laxiflora*. The restriction enzyme used to detect hybrid protocorms by cutting the ITS2 sequence is shown for each cross. The colored segments illustrate the ITS2 region of each species, while the number of enzyme cuts is shown visually.

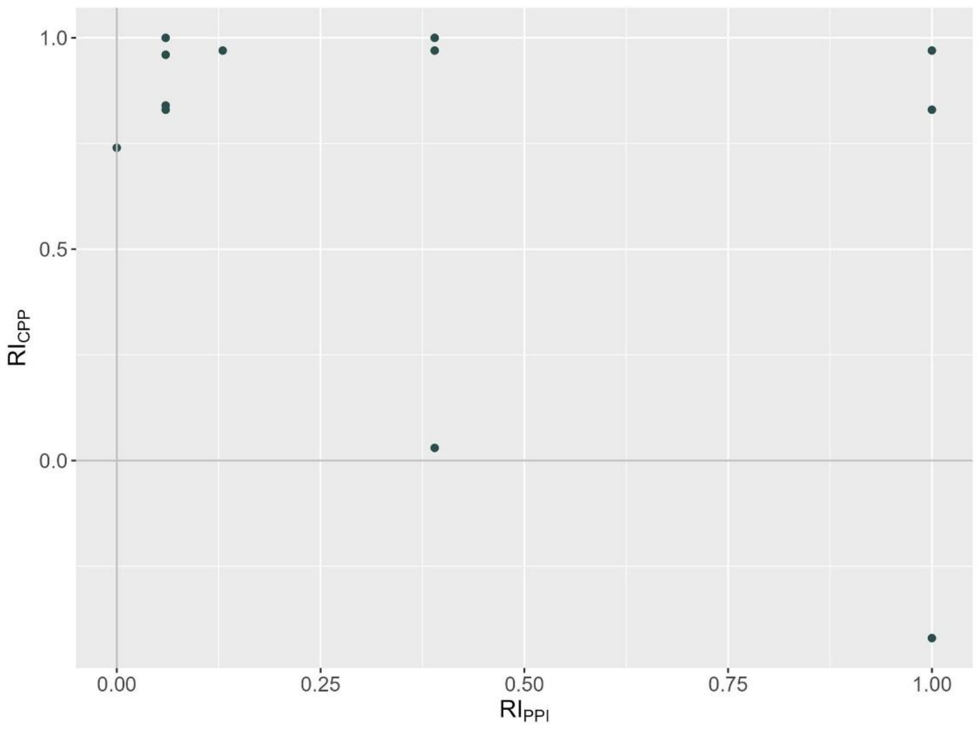


Fig. S2. Scatterplot showing the relationship between the two stages of post-pollination isolation. The x-axis represents pollen-pistil reproductive isolation ($RI_{\mathrm{PPI}}$), while the y-axis indicates CPP ($RI_{\mathrm{CPP}}$). Each point represents a different cross.

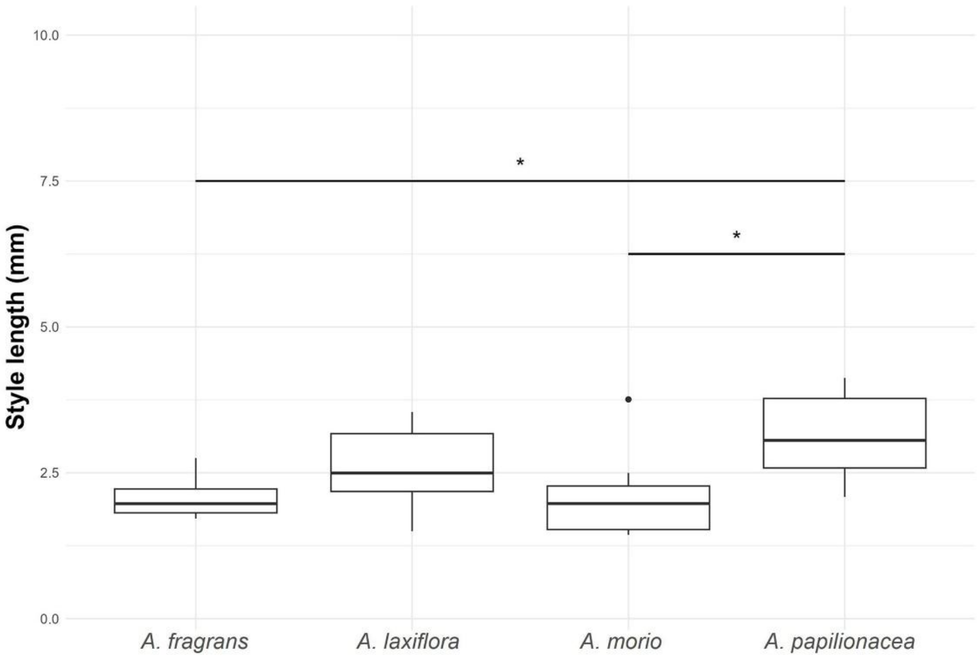


Fig. S3. Boxplot showing the style length (in mm) for the four species: *A. laxiflora*, *A. papilionacea*, *A. morio*, and *A. fragrans*. Each box represents the length distribution with the median (central line), interquartile range (upper and lower edges of the box), and extreme values (whiskers). Black horizontal lines and asterisks indicate significant differences between species pairs, based on Dunn's test with Bonferroni correction: $p \leq 0.05$ (*).
